# Exploring the relationships between sexual violence, mental health and perpetrator identity: a cross-sectional Australian primary care study

**DOI:** 10.1186/s12889-018-6303-y

**Published:** 2018-12-27

**Authors:** Laura Tarzia, Sharmala Thuraisingam, Kitty Novy, Jodie Valpied, Rebecca Quake, Kelsey Hegarty

**Affiliations:** 1 Department of General Practice, The University of Melbourne, 200 Berkeley Street, Carlton, Victoria 3053 Australia; 2 Centre for Family Violence Prevention, The Royal Women’s Hospital, Parkville, Australia

**Keywords:** Sexual violence, Mental health, Domestic violence/intimate partner violence, Sexual health, Women’s health, Primary care

## Abstract

**Background:**

Research supports the association between adult sexual violence (SV) and poor mental health. However, most studies focus on rape and physical sexual assault. Little is known about how more subtle forms of SV affect women’s well-being. Furthermore, evidence for the impact of the perpetrator’s identity is mixed. There is also little data from clinical populations to help health practitioners identify SV. This paper addresses these gaps by exploring the associations between different types of adult SV, perpetrator identity, and women’s mental health in the Australian primary care setting.

**Methods:**

We conducted a descriptive, cross-sectional study in general practice clinics. Adult women completed an anonymous survey while waiting for the doctor. Measures included PHQ-9 (depression), GAD-7 (anxiety) and PCL-C (post-traumatic stress disorder). SV was measured using items from the National Intimate Partner and Sexual Violence Survey and categorised into three groups (rape/sexual assault; coercive behaviours and/or reproductive control; and unwanted sexual contact).

**Results:**

We found significant associations between rape/sexual assault and poor mental health, and between coercion and/or reproductive control and higher PTSD and anxiety scores, compared to women with no SV experiences. SV perpetrated by an intimate partner was associated with significantly higher mean PTSD scores than SV perpetrated by a stranger, and significantly higher depression scores than SV perpetrated by another known person.

**Conclusion:**

Findings suggest that associations between SV and mental health are mediated by type of SV and perpetrator identity. Health practitioners should enquire about different types of SV beyond stranger rape as a cause of poor mental health, and about perpetrator identity to inform them about the likelihood of ongoing symptoms.

**Electronic supplementary material:**

The online version of this article (10.1186/s12889-018-6303-y) contains supplementary material, which is available to authorized users.

## Background

Sexual violence against women (SV) is globally prevalent [1]. In Australia, approximately one in five women have experienced some form of SV since the age of 15 years [2]. Research consistently shows strong associations between adult SV victimisation and poor mental health [3–5], including anxiety, depression, and post-traumatic stress disorder (PTSD) [5]. However, there is inconsistency around what behaviours are included in the definition of ‘sexual violence’ [6]. The majority of SV literature focuses only on rape or physically violent sexual assault [7], yet SV also encompasses other behaviours such as coercion, reproductive control, unwanted sexual contact, and forced consumption of pornography [4]. With the exception of a few studies examining the mental health impacts of coercion as an aspect of intimate partner violence [8, 9], and one study from the Cote d’Ivoire on reproductive control [10], little is known about the health effects of these more subtle types of SV, despite their prevalence [11].

In an earlier study [6] we found that poor mental health in women attending Australian general practices was associated with SV, even when a broader definition was used. Women most commonly reported experiencing public harassment or flashing, unwanted groping, and being coerced into having sex. Despite the more subtle nature of these incidents compared to rape or violent sexual assault, women who had experienced SV in our sample were significantly more likely than those who had not to experience higher levels of anxiety and depression, although the association with depression disappeared after controlling for childhood sexual abuse. In the present study, we sought to unpack this finding further by exploring the relationship between poor mental health and specific types of SV.

Another poorly understood relationship is that between the identity of a perpetrator of SV and women’s mental health. Studies consistently suggest that women are most likely to be sexually assaulted by a known perpetrator in their own home [12], yet community understandings of sexual violence typically focus on stranger rapes in dark alleyways. This is highly problematic, since compared to SV perpetrated by a stranger, SV at the hands of an intimate partner is associated with a higher risk of death or serious injury [13], exposure to multiple and repeated attacks [14], greater risk of sexually transmitted infection [15] and increased feelings of shame [16], all of which are likely to contribute to poor mental health outcomes. Despite this, findings around mental health and perpetrator identity have been mixed [17–20]. For instance, Ullman et al. [19] found no significant differences in mental health outcomes between women raped by a stranger and those raped by an intimate partner. On the other hand, Abrahams et al. [17] found higher rates of depression in women raped by a known perpetrator rather than a stranger, although they did not distinguish between an intimate partner and another known person. Several other studies also found worse mental health outcomes associated with SV perpetrated by a partner, including PTSD, stress and dissociation [18], and hyperarousal [20]. The majority of these studies, however, focus on rape or violent sexual assault, and several include only women who had already disclosed, or who were seeking help for SV.

As well as a paucity of knowledge around the circumstances of SV experiences and how these impact on women’s mental health, there is a lack of SV data from the primary care setting [6, 21, 22]. This is problematic given that the World Health Organization has recommended primary care as a key part of an effective early intervention and response [1]. The findings from our previous study suggest that almost half of women presenting to general practice clinics have experienced some form of SV [6], yet, health practitioners have little data from their own clinical population to guide practice.

The current paper aims to address these key gaps in the literature by reporting data from a cross-sectional study conducted in Australian general practice clinics. Our objectives are threefold: to explore the relationships between different types of sexual violence and mental health outcomes; to understand the relationship between perpetrator identity and mental health outcomes; and to provide data from a primary care sample to help guide practice. We argue that due to the complex relationship between women’s mental health and SV victimisation, general practitioners (GPs) and other health professionals ought to consider a range of circumstances and situations beyond those of stranger rape when seeing female patients with mental health symptoms.

## Methods

The present study built on previous work exploring the associations between women’s mental health and SV described elsewhere [6]. We used a short (10–15 min) anonymous survey with women aged over 18 years delivered via iPad or on paper in the waiting room of participating GP clinics.

### Practice recruitment

Information packs were posted to 101 private GP clinics in Victoria, Australia who had participated in a previous study on intimate partner violence [23]. A research assistant then telephoned the clinics a week later to follow up. The lead researcher attended interested clinics in person to speak with staff about the study. It was not a requirement that all doctors at a clinic agree participate in order for the clinic to be eligible.

### Participants

Eligible participants were adult women (> 18) waiting to see a participating doctor, who were able to provide informed consent, and had sufficient English comprehension to complete a survey. We needed a sample size of 336 women to have sufficient power to detect one third of a standard deviation in mental health scores between those who had experienced SV and those who had not. Based on our previous study [6], we assumed similar proportions in each group.

### Measures

SV experiences were determined using questions about ‘Sexual Violence Victimisation’ and ‘Control of Reproductive and Sexual Health’ taken from the US National Intimate partner and Sexual Violence Survey [11]. The items cover a broad range of behaviours including reproductive coercion, sex under the influence of alcohol or substances, rape and sexual assault, and unwanted kissing or touching. The identity of the perpetrator was asked using a single item developed for this study: ‘What was the relationship of the perpetrator to you?’. The answer options were: stranger, boy/girlfriend, a date, a partner living with you now, a partner you were living with at the time (now ex-partner), an ex-partner that you were not living with at the time, a family member, a friend, any other acquaintance. Severity of current depressive symptoms was measured using the PHQ-9 [24], a well-validated tool which has shown very good psychometric properties in primary care settings [24]. Respondents were asked to indicate on a 4-point scale how often they had experienced each of the 9 items in the previous 2 weeks. Scores range from 0 to 27, with a range of 5 to 9 suggesting mild depression, 10 to 14 moderate depression, 15 to 19 moderately severe depression and 20–27 severe depression. Anxiety severity was measured using the generalised anxiety scale (GAD-7) [25], a well-validated and widely-used tool. The GAD-7 has shown excellent psychometric properties in prior research [26]. Respondents were asked to indicate how often they had experienced each of the 7 items in the last 4 weeks: 0 = Not at all; 1 = Several days; 2 = More than half the days. Scores could therefore range from 0 to 14. Scores of 8 or above suggest possible presence of an anxiety disorder, when using the GAD-7 diagnostic algorithm for anxiety disorder, and scores of 5 or above suggest at least a mild level of anxiety. PTSD was measured using the PCL-C (civilian version) [27], a self-report tool with excellent test-retest reliability and high internal consistency. Scores for this measure range from 17 to 85. Although the PCL-C is not strictly a diagnostic tool, studies have suggested that a score of 30 is a good predictor of PTSD diagnosis in a primary care sample [28], and could therefore be considered ‘clinically meaningful’.

### Data collection

Data were collected between March and August 2016. Research assistants were placed in the clinic waiting rooms. All patients were informed that a research project was in progress. Female patients arriving for their appointment were asked by the practice manager or receptionist whether they would mind being approached whilst waiting for the doctor. If the woman declined, this was recorded along with her date of birth and postcode (with permission and for comparison purposes only). The research assistants were then notified so that they did not disturb the patient. Practice managers also alerted the researchers to any patients who would not be able to consent due to ill health/disability or lack of English comprehension. For safety reasons, any female patient closely attended by a male partner was not approached. Eligible female patients who agreed to be approached were provided with an information sheet outlining the study. The research assistants answered any questions or concerns in person. Women were then given an iPad or paper survey to complete. Written consent via a tick box was built into the survey on the initial screen of the iPad or first page of the paper version.

Survey responses collected on the iPad were saved to the Cloud as soon as the participant submitted the survey. Data collected using paper surveys were manually entered by a member of the research team. Ten percent of the paper survey data was cross-checked by a second researcher.

A number of strategies were put into place to maximise the safety and well-being of participants. These included: providing all women with resource cards on completion of their survey; ensuring that doctors were provided with information on responding to disclosures of SV and child abuse; ensuring that all team members were trained to respond sensitively to participant distress; and providing private spaces in which to speak with women if needed.

### Data analysis

STATA version 13.1 [29] was used for all analyses. Descriptive statistics were used to summarise participant demographics. SV items were categorised into three groups for analysis: 1. rape or sexual assault; 2. coercive behaviours and/or reproductive control without rape or sexual assault; 3. unwanted sexual contact only (see Table 1). Coercive behaviours and reproductive control were combined due to the similarities between the constructs. Perpetrator identities were collapsed into three categories: intimate partner, family member or other known person, and stranger. Linear mixed-effects models using restricted maximum likelihood estimation and random intercepts at the clinic level were used to estimate the mean difference in mental health score between those who had experienced a form of SV and those who had not. Outcome measures were adjusted by clinic (cluster) and experience of childhood sexual abuse, given that both these variables could potentially influence both adult SV victimisation [30, 31] and mental health [32–34]. Any participant characteristics that differed between those who experienced sexual violence and those who did not (see Table 2) were treated as potential confounders. These variables were tested in separate univariate models with the outcome variable, form of sexual violence and perpetrator identity. Only those variables found to be associated with both the outcome and independent variable were included as confounders in the adjusted model. Transformations for skewed continuous measures were considered. The distributions of residuals for each outcome were used to check the goodness of fit of the models and the influence of potential outliers on the regression models assessed. Under the linear mixed-effects regression model, missing data were assumed to be missing at random. Intra-clinic correlations for mental health outcomes were calculated using one-way analysis of variance.

**Table 1:** Sexual violence items

Item	Category
Has anyone ever:	
exposed their sexual body parts to you, flashed you, or masturbated in front of you?	Unwanted sexual contact
harassed you while you were in a public place in a way that made you feel unsafe?	
fondled or grabbed your sexual body parts?	
kissed you in a sexual way? Remember, we are only asking about things that you didn’t want to happen.	
made you show your sexual body parts to them? Remember, we are only asking about things that you didn’t want to happen.	
made you look at or participate in sexual photos or movies?	
When drunk, high, drugged or passed out and unable to consent, has anyone ever:	
had vaginal sex with you?	Rape/sexual assault
made you perform oral sex?	
forced you to receive anal sex?	
made you receive oral sex?	
Has anyone ever used physical force or threats of physical harm to make you:	
have vaginal sex with them?	Rape/sexual assault
try to make you have vaginal, oral, or anal sex with you, but sex did not happen?	
perform oral sex?	
let them put their fingers or an object in your vagina or anus?	
receive oral sex?	
receive anal sex?	
Have you ever had vaginal, oral or anal sex with someone after they pressured you by:	
wearing you down by repeatedly asking for sex, or showing they were unhappy?	Coercive behaviours and/or reproductive control
doing things like telling you lies, making promises about the future they knew were untrue, threatening to end your relationship, or threatening to spread rumours about you?	
using their authority over you, for example, your boss or your teacher?	
Have any of your romantic or sexual partners ever:	
refused to use a condom when you wanted to use one?	Coercive behaviours and/or reproductive control
tried to get you pregnant when you did not want to become pregnant; or tried to stop you from using birth control?

**Table 2:** Demographics by experience of sexual violence (*N* = 325)

	Sexual violence (*N* = 126)		No sexual violence (*N* = 179)	
		Missing		Missing
	n (%)	n (%)	n (%)	n (%)
Age^a^	50.5 (17.1)	–	57.8 (18.8)	1 (0.6)
Born in Australia	99 (78.6)	–	139 (78.5)	2 (1.1)
Aboriginal/Torres Strait Islander	1 (0.8)	2 (1.6)	–	3 (1.7)
Completed high school	99 (82.5)	6 (4.8)	115 (65.0)	2 (1.1)
Employed	75 (61.5)	4 (3.2)	88 (49.7)	2 (1.1)
Married/defacto	46 (36.5)	–	65 (38.2)	9 (5.0)
Lives alone	18 (14.3)	–	40 (23.5)	9 (5.0)

- represents 0 (0%)

20 (6.2%) with missing responses for sexual violence

^a^mean (standard deviation)

Sensitivity analyses using pattern-mixture models were carried out to test the robustness of the missing at random assumption. For data that is missing at random, the difference between the mean of the missing data and the mean of the observed data *δ*, is zero. The sensitivity analysis carried out in this study considers various plausible values for *δ* other than zero to simulate scenarios in which the missing data may not be missing at random. Positive values of *δ* indicate that participants with missing data have on average higher outcome scores than observed participants. Negative values of *δ* indicate that participants with missing data have on average lower outcome scores. In the sensitivity analyses, the regression analyses take into account the plausible values of *δ*. These regression results were compared with those from the main analysis to determine whether study conclusions changed if the missing data was not missing at random.

## Results

**Fig. 1:**
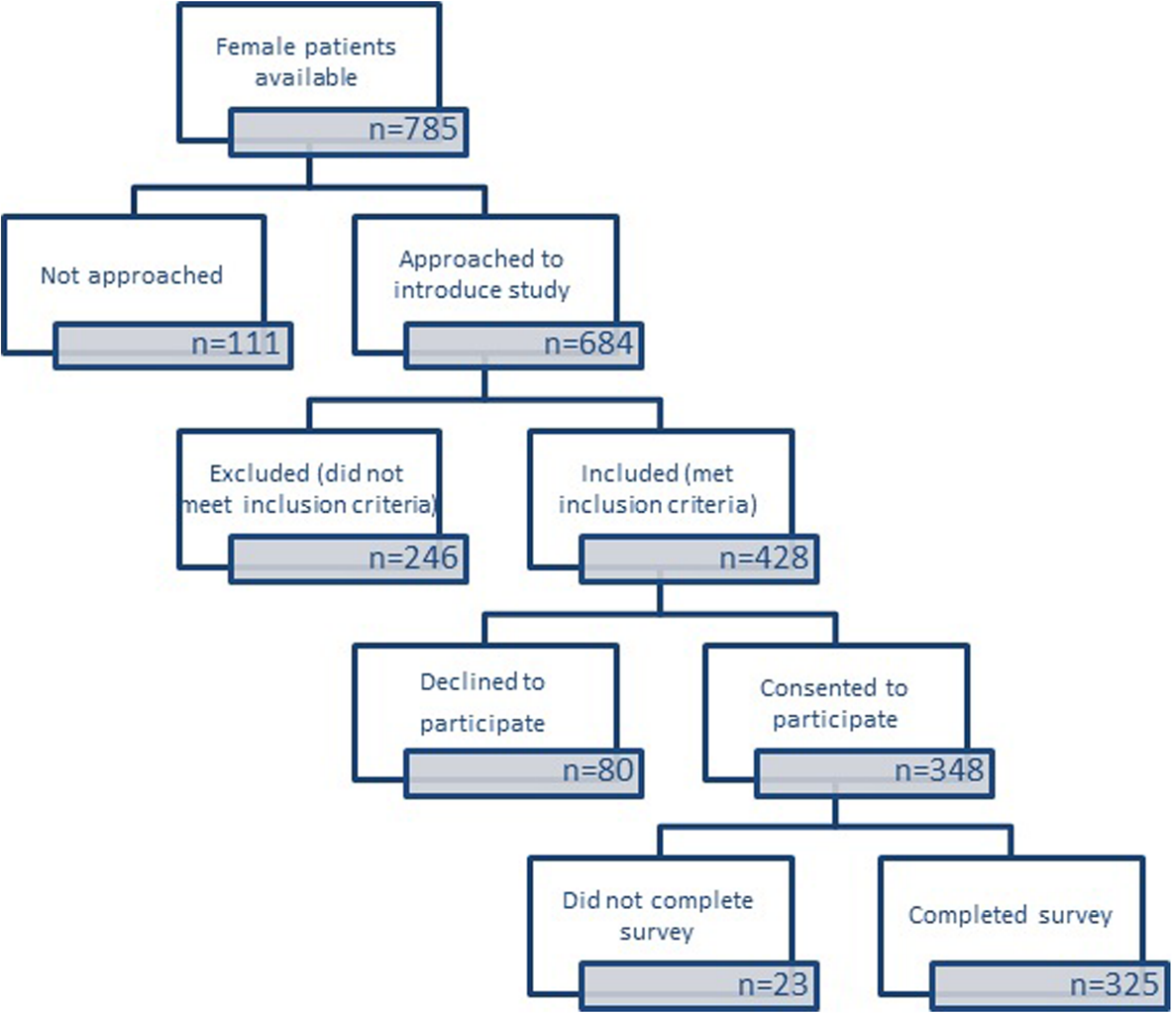
Recruitment of sample

Seven Victorian GP clinics were recruited, however, two dropped out due to lack of interest from doctors and one due to logistical issues. Four practices remained; two located in metropolitan Melbourne, one in an outer north-east suburb, and one in a coastal town in the Greater Geelong area. Thirty-two doctors were available across these clinics; 14 agreed to take part in the study. A total of 684/785 (87%) women were approached and asked to participate (see Fig. 1). Overall, 325/428 (76%) eligible women completed the survey, with between 7 and 125 women from each clinic (mean number of eligible participants per clinic = 81.3, SD = 53.5; intraclass correlations for PTSD = 0.01; depression =0.009; and anxiety = 0.01). Reasons for non-participation included: the woman being too unwell; needing to attend to children; discomfort with the topic, or having insufficient time to complete the survey.

Table 2 shows the demographics of the sample by experiences of SV. Twenty women did not provide data for the sexual violence items, leaving a total of 305 women included in our analyses. The majority of women were born in Australia, had at least completed high school and were employed.

Approximately 41% (*n* = 126/305) of participants had experienced some form of SV since the age of 15 years, and 11% (*n* = 34/305) had experienced it in the past 12 months. Sixteen percent (*n* = 48/305) had ever experienced rape or sexual assault. Overall, 17.5% (*n* = 53/305) had ever been coerced into having sex or experienced reproductive control. Seven percent (*n* = 22/305) had experienced coercion and/or reproductive control without also having experienced rape or sexual assault. Eighteen percent (56/305) of women had experienced only unwanted sexual contact such as groping, harassment, flashing or touching.

### Type of sexual violence experiences and mental health

All outcomes, PCL-C, PHQ-9 and GAD-7, demonstrated good internal consistency and reliability (Cronbach’s alpha 0.94, 0.88 and 0.76 respectively). There was a strong relationship between women’s experiences of rape or sexual assault and poor mental health (see Table 3). Women who had been raped or sexually assaulted had on average significantly higher PTSD scores (mean difference = 10.5, 95% CI =6.8 to 14.2) than women who had not experienced SV. These women also experienced significantly higher levels of anxiety (mean difference = 2.3, 95% CI = 1.1 to 3.5) and depressive symptoms (mean difference = 2.7, 95% CI = 0.9 to 4.4) compared to women with no SV experiences.

**Table 3:** Mental health outcomes by type of sexual violence (*N* = 325)

Mental health outcomes	Type of sexual violence	n	Mean score (SD)	Unadjusted		Adjusted^a^	
				Mean difference in mental health outcome score (95% CI)	*P*-value	Mean difference in mental health outcome score (95% CI)	*P*-value
PTSD	No experience of SV	175	23.7 (9.4)	–	< 0.001	–	< 0.001
	Unwanted sexual contact (only)	54	25.1 (9.3)	1.6 (−1.6 to 4.8)		0.8 (−2.5 to 4.0)	
	Coercive behaviour and/or reproductive control^b^	21	32.7 (11.9)	10.1 (5.3 to 14.9)		8.8 (3.9 to 13.7)	
	Rape/sexual assault (ever)	48	36.8 (14.4)	13.2 (9.9 to 16.5)		10.5 (6.8 to 14.2)	
Depression	No experience of SV	176	4.4 (5.0)	–	< 0.001	–	0.016
	Unwanted sexual contact (only)	54	4.8 (5.0)	0.4 (−1.1 to 1.9)		0.1 (−1.4 to 1.6)	
	Coercive behaviour and/or reproductive control^b^	22	6.2 (4.4)	2.0 (− 0.2 to 4.2)		1.5 (− 0.8 to 3.7)	
	Rape/sexual assault (ever)	48	8.3 (5.1)	3.9 (2.3 to 5.4)		2.7 (0.9 to 4.4)	
Anxiety	No experience of SV	176	2.4 (3.3)	–	< 0.001	–	< 0.001
	Unwanted sexual contact (only)	53	3.4 (3.4)	0.9 (− 0.1 to 1.9)		0.8 (− 0.3 to 1.8)	
	Coercive behaviour and/or reproductive control^b^	21	5.3 (3.3)	3.0 (1.5 to 4.5)		2.7 (1.2 to 4.2)	
	Rape/sexual assault (ever)	46	5.8 (3.7)	3.3 (2.2 to 4.4)		2.3 (1.1 to 3.5)	

20 (6.2%) participants had missing data for sexual violence

“No experience of SV” was used as the base level of the categorical exposure variable

^a^All models adjusted for clinic and experience of child abuse. PTSD and depression adjusted for age and employment status. Anxiety adjusted by age, employment status and whether participant had completed high school

^b^Includes those who have and have not experienced unwanted sexual contact

For women who had experienced coercive behaviour and/or reproductive control without rape or sexual assault, mean PTSD scores and mean anxiety scores were significantly higher compared to women who had not experienced SV (PTSD mean difference = 8.8, 95% CI = 3.9 to 13.7; anxiety mean difference = 2.7, 95% CI = 1.2 to 4.2). There was no statistical difference in mental health scores between those who experienced unwanted sexual contact alone and those who had not experienced sexual violence.

### Relationship between perpetrator identity and mental health

Seventy-eight of the 126 women who had experienced SV responded to the perpetrator identity question. For those who responded to this item, SV perpetrated by an intimate partner (see Table 4) was associated with significantly higher mean PTSD scores than SV perpetrated by a stranger (mean difference = 8.0, 95% CI = 0.8 to 15.2). Intimate partner sexual violence was also associated with significantly higher mean depression scores than women assaulted by another known person (mean difference = 2.8, 95% CI = 0.2 to 5.4). There was little difference in anxiety scores between perpetrator types. Despite approximately 37% missing data for perpetrator type, sensitivity analyses testing the robustness of the missing at random data assumption revealed no change in conclusions (see Additional file 1).

**Table 4:** Mental health outcomes by perpetrator type (*N* = 126)

Mental health outcomes	Perpetrator type	N	Mean score (SD)	Unadjusted		Adjusted^a^	
				Mean difference in mental health outcome score (95% CI)	*P*-value	Mean difference in mental health outcome score (95% CI)	*P*-value
PTSD	Intimate partner	30	36.8 (12.2)	–	0.098	–	0.086
	Stranger	21	28.1 (11.5)	−7.9 (− 15.1 to − 0.7)		− 8.0 (− 15.2 to − 0.8)	
	Known person	26	33.7 (15.7)	−2.9 (− 9.5 to 3.7)		− 4.7 (− 11.7 to 2.3)	
Depression	Intimate partner	30	8.4 (4.3)	–	0.187	–	0.070
	Stranger	21	5.9 (5.4)	−2.4 (− 5.2 to 0.3)		− 2.4 (− 5.2 to 0.3)	
	Known person	26	6.7 (5.6)	−1.7 (− 4.3 to 0.9)		−2.8 (−5.4 to − 0.2)	
Anxiety	Intimate partner	28	5.6 (4.0)	–	0.501	–	0.587
	Stranger	21	4.4 (3.3)	−1.2 (− 3.2 to 0.8)		−1.1 (−3.2 to 1.0)	
	Known person	25	5.0 (3.9)	−0.6 (−2.6 to 1.4)		−0.7 (− 2.9 to 1.4)	

48 (38%) had missing responses to perpetrator

“Intimate partner” was used as the base level of the categorical exposure variable

^a^Model adjusted for clinic and experience of child abuse

## Discussion

This cross-sectional study contributes to the knowledge base around women’s experiences of adult SV and provides a more nuanced view of the associations between different types of SV and mental health. It also highlights the long-lasting impacts of SV, which are present even when past childhood sexual abuse is taken into account. The study also provides data from a clinical population; there is a lack of such information currently available [21, 22] to guide practice and shape an effective response from health professionals.

Consistent with the existing literature [3, 5], women who had ever experienced rape or sexual assault had higher mean levels of PTSD, anxiety and depression compared to women who had not experienced SV. Furthermore, the mean PTSD score for women who had been raped or sexually assaulted was above the suggested threshold of 30 for screening in a primary care setting [28], and mean depression and anxiety scores for these women fell within the symptomatic range (mild to moderate symptoms). Whilst this was an expected finding, it is nonetheless important information for GPs and other primary care providers; as it highlights that historical SV may still contribute to current mental health symptoms in their clinical population.

A key finding of this study was that women who had experienced coercive behaviours or reproductive control (without also having experienced rape or sexual assault) had significantly higher PTSD and anxiety scores than women who had experienced no SV. Mean anxiety scores for these women were very similar to those of women who had been raped or sexually assaulted, and again, the mean PTSD score was above the suggested diagnostic threshold for a primary care sample. Given the small numbers of women in each group, reaching statistical significance suggests that the relationship between these behaviours and poor mental health is strong. This is a critical finding that has important implications for practice. General practitioners and other health professionals responding to women should explore past experiences of coercion or reproductive control – in addition to rape and sexual assault – as a possible factor in otherwise unexplained mental health symptoms, particularly PTSD and anxiety.

Our study also sheds light on the relationship between women’s mental health and the identity of the perpetrator. For those who responded to the perpetrator identify question, women assaulted by an intimate partner had significantly higher mean PTSD scores than women assaulted by a stranger and significantly higher mean depression scores than women assaulted by another known person such as a family member or friend. This supports the theory that SV perpetrated by an intimate partner has particularly serious impacts on women’s mental health. This may be due to the increased level of fear for personal safety that many women experience when living with a perpetrator of violence [12], as well as the often ongoing and frequent nature of the abuse. It is also likely that the breach of trust and sense of humiliation women may experience [35, 36] after intimate partner sexual violence may contribute to poor mental health outcomes. Despite this, intimate partner sexual violence is often neglected or assumed to be less serious than assaults perpetrated by a stranger. Clinicians ought to enquire about the identity of the perpetrator in order to inform themselves about the likelihood of ongoing mental health problems in the future.

### Limitations of the study

In the interests of keeping the survey brief, we were unable to capture data concerning the frequency of SV experiences, as well as other non-sexual traumas. Consequently, we have reported only associations rather than a causal relationship between SV and women’s mental health, which could only be achieved through a longitudinal study. Furthermore, the response rate from recruiting general practice clinics, although consistent with other studies [23], was low, and this may have affected the generalisability of the findings. Lastly, our sample size was not powered for three categories of SV, which led to low numbers in some of the groups. Many women also chose to skip the perpetrator question in the survey. This may account for the lack of statistical significance on some of the outcome measures, although for those where significance was reached it suggests a strong relationship.

## Conclusions

Our study found significant associations between women’s experiences of being coerced into having sex or having their reproductive autonomy taken away, and poor mental health outcomes. While the associations between rape/physical sexual assault and poor mental health have previously been identified within the literature, little was known about how more subtle forms of SV might impact on women’s well-being. The study also supports the theory that SV perpetrated by an intimate partner is particularly traumatic for women when compared to SV perpetrated by a stranger or another known person. These are important findings for health practitioners responding to women who present with otherwise unexplained mental health symptoms. This study represents an important first step in gaining a clearer picture of how SV impacts on women’s mental health; more research with a larger population group is recommended in order to explore these relationships further.

## Additional file

Additional file 1: Sensitivity analysis results. Results of sensitivity analyses using pattern-mixture models to test the robustness of the missing at random assumption. (DOCX 33 kb)

## Abbreviations

GAD-7: Generalized Anxiety Disorder 7 item scale
GP: General practitioner (family doctor)
PCL-C: Post-traumatic stress disorder checklist, civilian version
PHQ-9: Patient Health Questionnaire 9 item depression scale
PTSD: Post-traumatic stress disorder
SV: Sexual violence
