# Supplementary material for: Exploring the relationships between sexual violence, mental health and perpetrator identity: a cross-sectional Australian primary care study
Source: BMC Public Health. 2018 Dec 27;18:1410. doi: 10.1186/s12889-018-6303-y (PMC6307303; doi:10.1186/s12889-018-6303-y)
Supplement: Supplementary file 1 — Sensitivity analysis results. Results of sensitivity analyses using pattern-mixture models to test the robustness of the missing at random assumption. (DOCX 33 kb) [file 12889_2018_6303_MOESM1_ESM.docx]

**Appendix A: Sensitivity Analysis Results**
